# Karrikins: Regulators Involved in Phytohormone Signaling Networks during Seed Germination and Seedling Development

**DOI:** 10.3389/fpls.2016.02021

**Published:** 2017-01-24

**Authors:** Yongjie Meng, Haiwei Shuai, Xiaofeng Luo, Feng Chen, Wenguan Zhou, Wenyu Yang, Kai Shu

**Affiliations:** Key Laboratory of Crop Ecophysiology and Farming System in Southwest China (Ministry of Agriculture), Sichuan Engineering Research Center for Crop Strip Intercropping System, Institute of Ecological Agriculture, Sichuan Agricultural UniversityChengdu, China

**Keywords:** karrikins, ABA, GA, IAA, germination, photomorphogenesis

## Abstract

Seed germination and early seedling establishment are critical stages during a plant’s life cycle. These stages are precisely regulated by multiple internal factors, including phytohormones and environmental cues such as light. As a family of small molecules discovered in wildfire smoke, karrikins (KARs) play a key role in various biological processes, including seed dormancy release, germination regulation, and seedling establishment. KARs show a high similarity with strigolactone (SL) in both chemical structure and signaling transduction pathways. Current evidence shows that KARs may regulate seed germination by mediating the biosynthesis and/or signaling transduction of abscisic acid (ABA), gibberellin (GA) and auxin [indoleacetic acid (IAA)]. Interestingly, KARs regulate seed germination differently in different species. Furthermore, the promotion effect on seedling establishment implies that KARs have a great potential application in alleviating shade avoidance response, which attracts more and more attention in plant molecular biology. In these processes, KARs may have complicated interactions with phytohormones, especially with IAA. In this updated review, we summarize the current understanding of the relationship between KARs and SL in the chemical structure, signaling pathway and the regulation of plant growth and development. Further, the crosstalk between KARs and phytohormones in regulating seed germination and seedling development and that between KARs and IAA during shade responses are discussed. Finally, future challenges and research directions for the KAR research field are suggested.

## Introduction

Most angiosperm plants start a new stage of growth and development with seed germination. Although seed dormancy prevents germination, it is a very important approach for plant survival, especially under unfavorable conditions. Seeds can define whether the environmental conditions are appropriate for germination (Finch-Savage and Leubner-Metzger, 2006; Shu et al., 2015; Oracz and Stawska, 2016). For the dormant seeds, a series of environmental and endogenous signals co-occur to break seed dormancy, and then induce germination. Seedling establishment is another key stage of plant life cycle, which follows closely after germination. It is believed that well-developed seedlings result in well-developed plants (Finch-Savage et al., 2010; Imran et al., 2013).

Phytohormones play a dominant role in regulating seed germination and seedling establishment. Gibberellins (GA) can break seed dormancy and induce germination (Yamauchi et al., 2004), while abscisic acid (ABA) can promote seed dormancy and delay germination (Ali-Rachedi et al., 2004). Auxin [indoleacetic acid (IAA)] is also involved in regulating seed dormancy (Liu et al., 2013). Furthermore, IAA has been demonstrated to be an important regulator in the plant shade avoidance syndrome that adversely affects seedling development and crop yield (Casal, 2013a; Gommers et al., 2013; Procko et al., 2014). In addition to phytohormones, other chemical compounds have the ability to regulate plant growth and development, such as nitrogen oxide and reactive oxygen species (ROS), both of which have been demonstrated to regulate seed dormancy and germination (Bethke et al., 2006; Oracz et al., 2007, 2009; Oracz and Karpiński, 2016).

In 2004, chemists purified 3-methyl-2*H*-furo [2, 3-*c*] pyran-2-one from the smoke of burning plant material (Flematti et al., 2004). Subsequently, several analogs to 3-methyl-2*H*-furo [2, 3-*c*] pyran-2-one were found and collectively named as karrikins (Flematti et al., 2007; Dixon et al., 2009). Subsequent studies revealed that KARs have significant biological activities in promoting germination and seedling establishment of model plant *Arabidopsis* (Waters and Smith, 2013; Flematti et al., 2015). KARs may regulate seed germination and shade responses by interacting with endogenous phytohormones signaling networks. In this review article, the relationship between KARs and SL is summarized, and then we discuss the mechanisms through which KARs interact with different phytohormones, and the crosstalk among KARs, ABA, GA, and auxin in the processes of germination and early seedling establishment. Finally, the challenges and research directions in the following study of KARs research field are suggested.

## The Relationship Between KARs and SL

So far, six different isoforms of KARs family are documented, KAR_1_–KAR_6_; and all of which contain a five-membered butenolide ring and a six-membered pyran ring (Dixon et al., 2009; Flematti et al., 2009; Waters et al., 2014). The primary difference among KARs family members is the number and location of methyl group(s) (Flematti et al., 2009). Interestingly, the butenolide moiety of KARs has high similarities with the D-ring of SL, a compound which is synthesized and exuded from roots, and also triggers the germination of parasitic weeds (Yoneyama et al., 2007; Dor et al., 2010; Waters et al., 2012).

**FIGURE 1:**
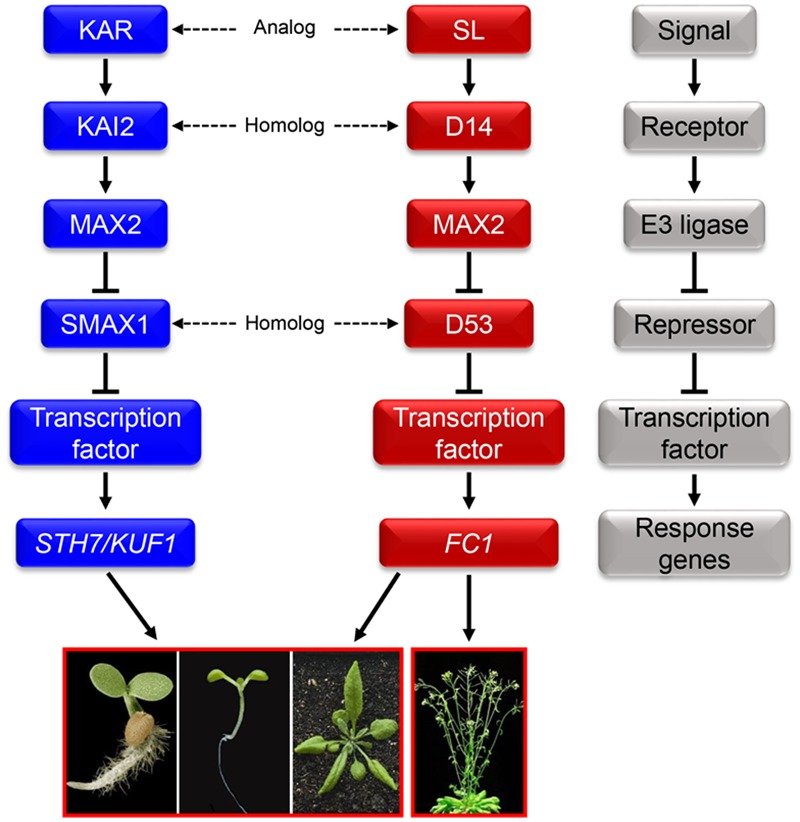
**The relationship between karrikins (KARs) and strigolactone (SL).** Proposed signal transduction of KARs and SL mediated by KAI2 and D14, and MAX2. The conformations of KAI2 and D14 will change once bound with KARs and SL, respectively. The conformational change allows KAI2 to interact with MAX2 to form a SCF E3 ubiquitin ligase complex which can degrade the repressor SMAX1/SMAX1-LIKE. Following, the activated transcription factor can regulate the expression of KARs response genes. SL binds to the receptor D14 and then MAX2 to form a SCF E3 ubiquitin ligase complex as well. Then the repressor D53 is degraded, helping the SL signal to transduct successfully to SL response genes. High similarities exist between the KARs and SL signaling pathways: KARs and SL are analogs; KAI2 and D14 are homologs; MAX2 is a communal F-box protein; SMAX1 and D53 are homologs (Nelson et al., 2010; Jiang et al., 2013; Waters et al., 2013; Zhou F. et al., 2013; Bennett and Leyser, 2014; Smith and Li, 2014). The gray block schemes shows the common signaling pathway model of KARs and SL which contains signals, receptors, E3 ubiquitin ligase, repressors, transcription factors and response genes.

Due to the significant promotion effect of KARs on seed germination of some species, the detailed mechanisms of KARs signaling has always been one of the most written topics in this field. KARRIKIN INSENSITIVE2 (KAI2) is the receptor in the signaling pathway of KARs (**Figure 1**) (Waters et al., 2013). When bound by KARs, KAI2 undergoes conformational changes (Guo et al., 2013; Zhao et al., 2013). Subsequently, KARs and KAI2 might form a SCF E3 ligase complex with MORE AXILLARY GROWTH2 (MAX2) (Waters et al., 2012, 2013; Kagiyama et al., 2013). The SCF complex can then promote the degradation of SMAX1 which is a repressor in KARs signaling pathway (Stanga et al., 2013). Further, other repressors of the KARs signaling pathway have been documented and named as SMAX1-LIKEs (Stanga et al., 2013). It is noted that the various repressors involved in its signaling pathway lead to the diversiform biological functions of KARs (Smith and Li, 2014).

As well as the similarities in chemical structures between KARs and SL, many components of these two signaling pathways are analogs or homologs (**Figure 1**) (Flematti et al., 2015; Morffy et al., 2016). Firstly, both signaling pathways are composed of receptors, E3 ligases and signal repressors; secondly, the receptor of SL signal, AtD14, is a homolog of KAI2 which is the receptor of KARs (Kagiyama et al., 2013; Waters et al., 2013); thirdly, the repressor of SL signaling pathway, D53, is also a homolog of SMAX1, the repressor in KARs signaling transduction pathway (Jiang et al., 2013; Zhou F. et al., 2013).

The similar chemical structures and signaling pathways of KARs and SL suggest common biological functions. Extensive studies reveal that both KARs and SL can promote seed germination and inhibit hypocotyl elongation (Nelson et al., 2010; Waters and Smith, 2013). However, the delicate distinctions between KARs and SL can result in some differences in other biological functions. For example, AtD14 cannot replace KAI2 during the processes of seed germination and seedling development; while KAI2 cannot take the place of AtD14 to regulate branch formation (Waters et al., 2015). Furthermore, GR24, a synthetic analog of SL, could not promote the expansion of cotyledons, while KARs could (Nelson et al., 2010); and GR24 could repress shoot branching, but KARs could not (Fukui et al., 2011; Nelson et al., 2011; Boyer et al., 2012). These striking differences in bioactivities of the two structurally similar butenolide compounds imply two distinct response systems in plants (Scaffidi et al., 2013), although the detailed components and precise mechanisms still need further dissection.

The signaling pathway mode of KARs and SL is very important and generally occurs in phytohormones signaling transduction, such as GA, IAA, and salicylic acid (Schwechheimer, 2008; Xu et al., 2010; Sun, 2011; Van der Does et al., 2013). If the signal is weak or absent, the receptors remain dormant as well as the E3 ubiquitin ligase, and subsequently the repressors repress the transcription of response genes. However, in the presence of signals, the activated E3 mediates the degradation of repressors and release the expression of response genes to regulate plant growth and development.

## KARs Regulate Germinability of Seeds by Interacting with Phytohormones in *Arabidopsis*

Seed dormancy and germination are not only important to plants but also to human beings, since germination rate is one of the main determinants that affects production in agriculture systems. KARs induce seed germination under weak light conditions by enhancing the response of seeds to light (Drewes et al., 1995; Nelson et al., 2010), whereas the acceleration effect of KARs on germination disappears in dark conditions (Nelson et al., 2010). However, fresh *Arabidopsis* seeds are insensitive to KARs, but the seeds become sensitive to KAR treatment after the after-ripening treatment (Waters et al., 2014). Further, the acceleration effect of KARs on seed germination also depends on *Arabidopsis* ecotype and depth of seed dormancy (Nelson et al., 2009).

**FIGURE 2:**
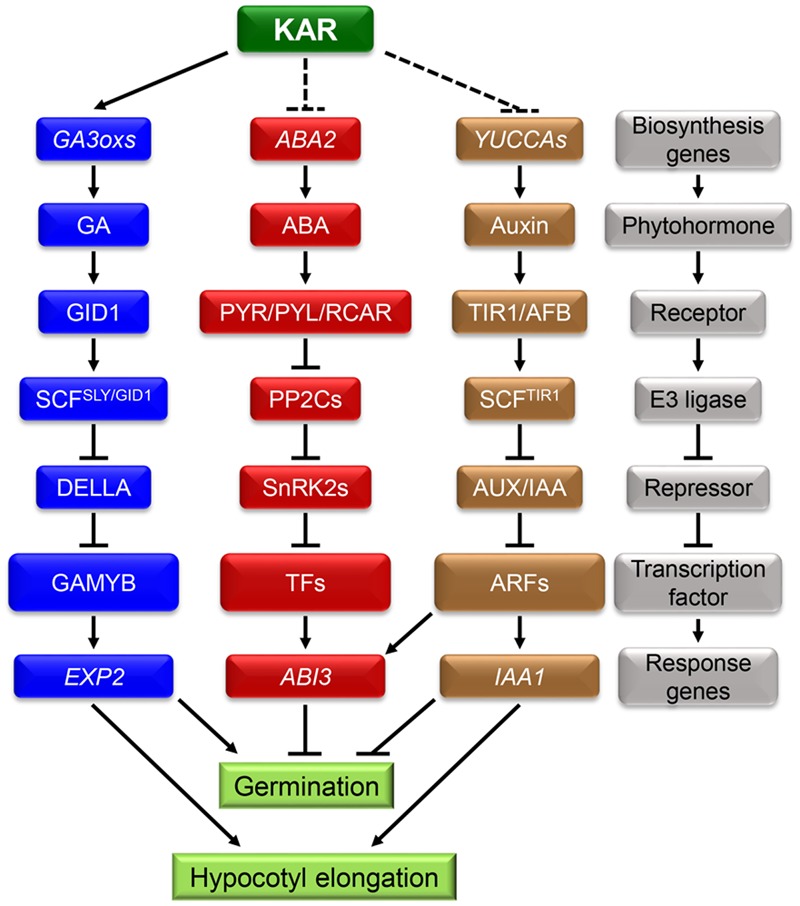
**Karrikins may regulate seed germination and hypocotyl elongation by affecting endogenous phytohormones crosstalk.** A hypothesis about the interaction between KARs and endogenous phytohormones: KARs can accelerate seed germination by enhancing GA biosynthesis. At the same time, KARs may inhibit the signals of ABA and IAA. In the following process of seedling establishment, KARs showed significant promotion effect and the expression of *IAA1* was down-regulated. It indicates that KARs may promote seedling establishment by inhibiting the IAA signaling pathway. In the gray block schemes, the components of the three phytohormones signaling pathway are showed. They are all composed of biosynthesis genes, phytohormones, receptors, E3 ligases, repressors, transcription factors and response genes.

During the process of germination, subtle changes in environmental conditions can be sensed by seeds and these cues can further affect internal signals such as phytohormones signaling networks. Numerous studies demonstrated that ABA and GA antagonistically regulate seed germination (Daws et al., 2007). ABA induced seed dormancy and inhibited germination, while GA had converse effects on those processes (**Figure 2**) (Xi et al., 2010). Consequently, the ratio of ABA/GA had a decisive and critical effect on the process of seed germination (Seo et al., 2006; Shu et al., 2013; Meng et al., 2016). In conclusion, the dynamic balance between ABA and GA has a unique role in regulating seed dormancy and germination (Finkelstein et al., 2008). Recent studies showed that IAA can also regulate seed dormancy and germination (**Figure 2**). Exogenous IAA effectively inhibited the pre-harvested sprouting of wheat spikelets (Ramaih et al., 2003). Furthermore, the process of germination was strongly inhibited in the transgenic plants *iaaM-OX* which possess higher levels of IAA in seeds (Cheng et al., 2006). On the contrary, the mutation in IAA biosynthesis genes *YUCCAs* led to lowering seed dormancy level (Liu et al., 2013). All of this evidence indicates that IAA has an important role in promoting seed dormancy and inhibiting germination.

Since ABA, GA and IAA are all involved in regulating seed dormancy and germination, a hypothesis that there is an interaction between KARs and those phytohormones needs to be further investigated. A former study showed that ABA removes the acceleration effect of KARs on germination and KARs need the biosynthesis of GA to promote seed germination (Nelson et al., 2009). In addition, KARs treatment promoted the expression of GA biosynthesis genes *GA3ox1* and *GA3ox2* (Nelson et al., 2010), but no transcription evidence is investigated about that of ABA thus far.

Indoleacetic acid regulates seed dormancy and germination by mediating the signal of ABA. AXR2 and AXR3 are transcriptional repressors of IAA signaling pathway (Sabatini et al., 1999; Nagpal et al., 2000). The seeds of *axr2-1* and *axr3-1*, which have a weaker endogenous IAA signal, are insensitive to ABA; but the blocking-up of IAA signaling pathway has no effect on the endogenous ABA content (Liu et al., 2013). On the contrary, *aba2*, the ABA deficient mutant, did not affect seed sensitivity to IAA. These results suggest synergistic effects between IAA and ABA in the process of germination. Subsequent investigations showed that the seeds of *abi3-1* mutant germinate normally in the presence of exogenous IAA and ABA (Liu et al., 2013). Furthermore, during the process of seed imbibition, *ABI3* transcription remained at a higher level in *iaaM-OX* transgenic plants, compared to wild type. On the contrary, the *ABI3* mRNA content in *arf10 arf16* seeds decreased gradually during imbibition (Liu et al., 2013). It indicates that ABA can regulate seed germination and dormancy in an IAA-dependent manner. Further, ABA can positively regulate seed dormancy by inhibiting GA signaling and working synergistically with IAA; GA and IAA may therefore regulate seed dormancy antagonistically. But the precise mechanism underlying the synergy effect between ABA and IAA, and antagonism between GA and IAA remains elusive so far.

Importantly, KARs suppress the expression of *IAA1* which is IAA response genes (Yang et al., 2004; Nelson et al., 2011; Gilkerson et al., 2015). Furthermore, as the analog of KARs, SL could regulate shoot branching by triggering the degradation of PIN1 which determines the polar transportation of IAA (Petrasek et al., 2006; Shinohara et al., 2013). Accordingly, KARs may accelerate seed germination by suppressing the signals of IAA. Whether KAI2 and MAX2 are also involved in the interactions between KARs and phytohormones during seed germination still needs further investigation. Furthermore, both KARs and IAA interact with ABA during germination, thus whether KARs affect ABA signal by regulating IAA signaling pathway is still unknown.

## The Effect of KARs on Crop Seed Germination

Most investigations about the acceleration effect of KARs on seed germination have been focused on the model plant *Arabidopsis* (Nelson et al., 2010; Waters et al., 2013) and the fire-following species (Keeley and Pizzorno, 1986; Daws et al., 2007). Subsequent investigations revealed that many weed seeds, even some horticultural crop seeds such as lettuce (*Lactuca saliva*) and tomato (*Lycopersicon esculentum*) were responsive to KARs (Drewes et al., 1995; Jain et al., 2006; Stevens et al., 2007). Can KARs be applied to regulate seed germination of crops? A recent study has demonstrated that KARs delayed soybean seed germination by enhancing ABA biosynthesis and impairing GA biogenesis (Meng et al., 2016). Surprisingly, KARs only inhibited soybean seed germination under shade conditions, rather than white light and dark conditions, which is completely distinct from the effect of KARs in *Arabidopsis*. Quantification of phytohormones showed that KARs enhanced ABA biosynthesis while impairing that of GA, and subsequently resulted in the decrease of GA_4_/ABA ratio. The following evidence including transcription patterns of ABA and GA metabolic related genes and inhibitors of ABA biosynthesis was consistent with the phenotype and hormone quantification (Meng et al., 2016). In conclusion, KARs delay soybean seed germination by regulating the ratio of GA/ABA under shaded conditions. Apart from soybean, the germination of other species such as *Capsella bursa-pastoris, Bromus sterilis*, and *Alopecurus myosuroide* could also be inhibited by KARs (Daws et al., 2007), but the detailed mechanisms still need further dissection.

Why do KARs repress seed germination in some species, such as soybean? It is noted that soybean originates in China, a non-Mediterranean climate region. It was suggested that the difference of environment may result in different response mechanisms in the evolution history (Meng et al., 2016). Secondly, cultivated soybean is artificially bred. Compared with the wild soybean, some critical genes might encounter deficiency or mutation which would also result in a distinct response mechanism to KARs treatment (Meng et al., 2016).

Though KARs did not show any acceleration effect on seed germination in soybean, KARs may still have applications in agricultural production. For example, treating field soil with KARs may cause “suicidal germination” of agricultural weeds so that the weeds can be eliminated easily (Flematti et al., 2015). Pre-harvest sprouting of soybean, especially under high temperature and humidity conditions, has an extremely negative impact on seed yield and nutritional quality (Quinhone and Ida, 2015; Shu et al., 2015). Based on the inhibition effect of KARs on soybean seed germination, spraying the KARs solution on mother plants in natural field may decrease pre-harvest sprouting of soybean. In future work, the effect of KARs on seed germination of other crops such as wheat, rice and maize still needs further analysis.

## Shade: Potential Application of KARs

Seedling development is another critical phase in the plant life cycle (Eastmond et al., 2015). In natural plant community or agricultural system, vegetation canopy decreases the red/far red light ratio and light intensity sensed by lower blades (Franklin, 2008; Casal, 2012). Shade affects almost all stages of growth and development of plants, including seed germination, seedling development and stem elongation (Valladares and Niinemets, 2008; Casal, 2013b). In most cases, the effects of shade are undesirable, including excessive growth and lower resistance to biotic and abiotic stresses (Kobata et al., 2000; Ballaré et al., 2012; Wit et al., 2013). In the face of shade stress, plants have evolved two completely distinct response mechanisms: shade tolerance and shade avoidance (Gommers et al., 2013).

Current studies showed that KARs could enhance the sensitivity of seedlings to light and promote seedling establishment (Waters and Smith, 2013). The hypocotyl was greener in KARs treatment and the chlorophyll content was higher; furthermore, the elongation of the hypocotyl was inhibited by KARs; and these promotion effects were independent of the plant genetic background (Nelson et al., 2010). Since KARs are so helpful in the regulation of photomorphogenesis by inducing sensitivity of seedlings to light, it is hypothesized that KARs may be an efficient solution to attenuate plant shade avoidance syndrome.

## KARs may Interact with IAA to Eliminate Shade Response

Phytohormones always play an efficient role in regulating the shade response of plants. Numerous studies showed that IAA, GA and brassinolide induce the elongation of hypocotyl by promoting cell elongation (Lilley et al., 2012; Chen et al., 2013; Bernardo-García et al., 2014; Oh et al., 2014). Further, both GA and brassinolide regulate hypocotyl growth in an IAA-dependent manner (Stamm and Kumar, 2010; Chapman et al., 2012; Zhou X.-Y. et al., 2013). Consequently, IAA appears to be the most dominant regulator in shade avoidance response regarding plant hypocotyl elongation.

On one hand, IAA content in the hypocotyl significantly increased under shade conditions; on the other hand, mutants deficient in IAA biosynthesis were insensitive to shade stress (Stone et al., 2008; Tao et al., 2008; Keuskamp et al., 2010; Cole et al., 2011). A further study in *Brassica rapa* also showed that, the excess IAA was biosynthesized in the cotyledons and transported to the hypocotyl under shade conditions (Procko et al., 2014). In conclusion, shade stress may regulate hypocotyl elongation mainly by promoting the biosynthesis and transportation of IAA. As KARs repressed the expression of *IAA1* (**Figure 2**) (Nelson et al., 2011), thus a hypothesis is proposed: the biosynthesis and transport of IAA in the process of seedling establishment may be inhibited by KARs. But whether KARs promote seedling establishment by inhibiting the IAA signaling pathway still needs more investigation.

As a critical factor in KARs signaling pathway, *MAX2* may have important roles in regulating germination and seedling development. *max2* mutants showed deep seed dormancy, epinastic leaves and long hypocotyls under white light, red light, far-red light, and blue light conditions (Nelson et al., 2011; Waters et al., 2012; Stanga et al., 2013; Jia et al., 2014). This suggests that *MAX2* is a positive regulator of photomorphogenesis (Shen et al., 2007). Consequently, the relationship between *MAX2* and the light signaling pathway needs further investigation. As a negative regulator in light signaling pathway, quadruple mutant of *PIF* (*pifq*) showed enhanced germination and seedling establishment under both dark and red light conditions (Shin et al., 2009). The seeds of double mutant between *pif1* and *max2* showed an intermediate germination rate phenotype. Further, the double mutants showed the similar phenotypes of hypocotyl length to *max2*, which indicated that *MAX2* is epistatic to *PIF* (Shen et al., 2012). But the specific relationship between *PIF1* and *MAX2* in the process of seedling establishment is still unclear. Interestingly, the de-etiolation phenotype of *cop1* could be partially suppressed by *max2*, while hypocotyl elongation in *max2* could be suppressed by *cop1*. This result suggests *COP1* may be parallel or epistatic to *MAX2* (Shen et al., 2012). As a positive regulator of photomorphogenesis, *HY5* acts downstream of multiple photoreceptors. But the hypocotyl length of *hy5max2* was significantly longer than both *hy5* and *max2* which suggests that *MAX2* regulates KARs and SL responses independently of *HY5* (Waters and Smith, 2013). The evidence mentioned above indicated that *MAX2* has an interaction with light signaling pathway, but the specific mechanisms still need more investigation.

In addition to the interaction of *MAX2* with light signaling pathway, the relationship between *MAX2* and phytohormones has also been investigated. IAA up-regulated the expression of SL biosynthesis genes, and the latter repressed the transportation of IAA in a MAX2-dependent manner (Foo et al., 2005; Hayward et al., 2009). This evidence suggests that *MAX2* may be involved in regulating IAA transportation. Subsequent studies also showed that *MAX2* can suppress the IAA transport by inhibiting the transcription of *PIN* genes which regulate IAA transportation (Bennett et al., 2006; Nodzynski et al., 2016). Consequently, increased IAA transportation in *max2* contributes to the long hypocotyl phenotype (Shen et al., 2012). Furthermore, *MAX2* also regulated the biosynthesis of ABA and GA to affect seed germination positively (Shen et al., 2012). Based on the evidence described above, MAX2 is involved in the crosstalk of phytohormones to regulate seed germination and photomorphogenesis.

KARRIKIN INSENSITIVE2 was initially named as *HYPOSENSITIVE TO LIGHT* (*HTL*) (Sun and Ni, 2011). Like *max2, kai2* mutants also showed a long hypocotyl phenotype (Waters et al., 2012). Interestingly, the double mutant *hy5kai2* showed longer hypocotyl compared to *hy5* and *kai2*, which is similar to that of *hy5max2* (Waters and Smith, 2013). It indicates that *KAI2* regulates seedling establishment independently of *HY5*. But the relationship of *KAI2* with other photomorphogenesis regulating factors such as *PIFs* and *COP1* still needs more research.

## Conclusion and Future Perspectives

The studies discussed above show that KARs can regulate seed germination and seedling development by regulating the crosstalk among endogenous phytohormones such as ABA, GA, and IAA. For intensively understanding the relationship among KARs and these phytohormones, there are still remaining important questions to be dissected.

Although ABA and IAA can induce seed dormancy synergistically while GA and KARs can accelerate germination, there is still no direct evidence that shows that KARs can affect the content or signaling pathway of IAA during the process of seed germination. The specific mechanism underlying KARs regulating endogenous phytohormones during seed germination is still unclear, especially for IAA. Furthermore, KARs could promote seedling development of *Arabidopsis* and inhibit the expression of *IAA1* (Nelson et al., 2011). Thus, whether this promotion effect is due to the suppression effect of KARs on the IAA signaling pathway still needs further investigation. This hypothesis will be valuable for modern agriculture systems which suffers yield loss from shade avoidance response.

In addition to phytohormones, ROS is also involved in regulating seed dormancy and germination. The ability to interact with lipids, DNA and protein molecules in the cell makes ROS an important regulator during seed germination (Oracz et al., 2007; Oracz and Karpiński, 2016). Since ROS continuously exists in the processes of seed development stages and during storage (Pukacka and Ratajczak, 2005; Bailly et al., 2008; Leymarie et al., 2012), the phytohormones such as ABA and GA have been demonstrated to interact with ROS in regulating seed germination (Oracz and Karpiński, 2016; Shu et al., 2016). Therefore, whether KARs can regulate seed germination by interacting with ROS mediated by ABA or GA is an interesting hypothesis.

In terms of regulating seed germination, the distinct species originated from different areas might lead to different responsiveness to KARs. However, whether KARs have the similar or distinct effects on wild cultivars and cultivated cultivars within one species still needs more investigation, especially in crops species including wheat, maize and rice.

Finally, it is noted that *kai2* shows a similar hypocotyl elongation phenotype to *max2*, which has increased IAA transportation (Waters et al., 2012). Whether there is an increase of IAA transportation in *kai2* is still unknown. Consequently, whether *KAI2* is also involved in the IAA signaling pathway just like *MAX2* needs more investigation. Further, as positive photomorphogenesis regulators, both *KAI2* and *MAX2* show an interaction with the light signaling pathway which is incompletely understood. Furthermore, recent studies showed that KAI2 may perceive non-KARs signals (Conn and Nelson, 2015; Waters et al., 2015). Therefore, the impaired photomorphogenesis phenotypes of *kai2* and *max2* suggests a possible signaling pathway which is independent of KARs, but KARs can enhance the signaling outputs by interacting with the signaling networks of different phytohormones.

## Author Contributions

Conceived and designed the manuscript: KS and WY. Analyzed KAR and SL relationship: KS, YM, HS, and XL. Analyzed KAR regulates seed germination: YM, HS, FC, and WZ. Analyzed KAR involved in shade response: KS, FC, and WZ.

## Conflict of Interest Statement

The authors declare that the research was conducted in the absence of any commercial or financial relationships that could be construed as a potential conflict of interest.
